# Spinel ZnFe_2_O_4_ Nanoparticles Doped with Ba^2^^+^ for High-Performance Cu(II) Extraction via *d*‑SPE–FAAS

**DOI:** 10.1021/acsomega.5c07865

**Published:** 2025-10-28

**Authors:** Dilges Baskin

**Affiliations:** Muradiye Vocational School, Chemistry and Chemical Processing Technologies Department, 53000 Van Yuzuncu Yil University, Van 65080, Turkey

## Abstract

In this study, a dispersive solid-phase extraction (*d*-SPE) method was developed and characterized using Ba-doped ZnFe_2_O_4_ spinel nanoparticles for the selective preconcentration and determination of Cu­(II) ions in environmental- and food-based matrices. The structural features of the nanosorbent were thoroughly investigated using SEM, SEM–EDX, XRD, and FTIR techniques. The integration of Ba^2^^+^ into the spinel lattice structure enhanced the adsorbent’s surface reactivity, and the material is therefore presented as a high-surface-area spinel sorbent, which contributed to the efficient and selective retention of Cu­(II) ions. Following the optimization of extraction parameters (pH 9.0, 40 mg of sorbent, 0.3 mL of HNO_3_, 60 s vortex for adsorption, and ultrasonic mixing for desorption), quantification was carried out using flame atomic absorption spectrometry (FAAS). The method exhibited excellent analytical performance, achieving a limit of detection (LOD) of 0.67 ng mL^–^^1^, a wide linear dynamic range of 5.0–300 ng mL^–^^1^, and a correlation coefficient (*R* ^2^) of 0.9992. The developed *d*-SPE–FAAS method achieved an LOD improvement factor of 62, along with an enhancement factor of 29 and a classical preconcentration factor of 30. In addition, a Langmuir isotherm study at pH 9.0 indicated a maximum adsorption capacity of 104.2 mg g^–^^1^, confirming the affinity of the Ba-doped ZnFe_2_O_4_ sorbent for Cu­(II). Applicability of the method was evaluated using real samples, including green tea infusions, tap water, and domestic wastewater obtained from the General Directorate of VASKİ (Van Water and Sewerage Administration). Quantitative recoveries ranging from 94% to 106% were obtained in real matrices, demonstrating the method’s accuracy and reliability in complex sample compositions. The developed *d*-SPE–FAAS protocol provides a simple, sensitive, and cost-effective approach for determining trace levels of Cu­(II), exhibiting strong potential for routine copper monitoring in both environmental and food-derived samples.

## Graphical abstract

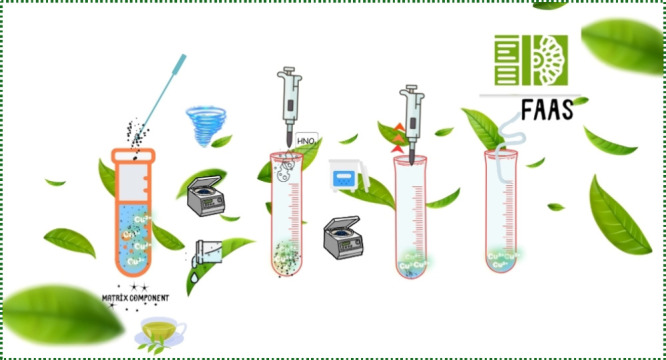

## 1 Introduction

Spinel ferrite nanoparticles, with the general formula MFe_2_O_4_, have emerged as promising candidates in analytical chemistry due to their thermally stable cubic lattice and chemically adaptable surface properties.^1^ Among these, zinc ferrite (ZnFe_2_O_4_) is classified as a normal spinel, in which Zn^2^^+^ occupies tetrahedral sites and Fe^3^^+^ resides in octahedral sites within the oxygen framework.^1^ Interestingly, at the nanoscale, partial inversion of cation sites may occur, leading to ferrimagnetic behavior that contrasts with the antiferromagnetic nature of bulk ZnFe_2_O_4_. This cation redistribution, alongside the abundance of surface hydroxyl groups, enhances the surface reactivity and adsorption potential of the ZnFe_2_O_4_ nanoparticles. As high-surface-area spinel sorbents with strong physicochemical stability, they offer promising potential for separation-based applications.^2^

The adsorptive performance of ZnFe_2_O_4_ arises from its ability to bind metal ions via surface hydroxyl coordination and electrostatic interactions, particularly under nearly neutral pH conditions. These characteristics are especially beneficial for trace metal preconcentration in complex matrices. Recent literature has demonstrated the potential of ZnFe_2_O_4_ and its nanocomposites in sample pretreatment, including heavy metal adsorption and dispersive solid-phase extraction (*d*-SPE) protocols. For instance, Chen et al. prepared magnetic ZnFe_2_O_4_ nanotubes and applied them in micro-*d-*SPE for Co­(II), Ni­(II), Mn­(II), and Cd­(II), achieving quantitative retention followed by effective elution with diluted nitric acid.^2b^ The sorbent showed optimal performance in the pH 6–8 range, which is ideal for selective metal binding.^2b,3^ Other nanohybrids, such as Ba-doped ZnFe_2_O_4_, have previously been employed in various applications, including as magnetically separable adsorbents (e.g., Ba^2^^+^–ZnFe_2_O_4_/reduced graphene oxide nanohybrids for dye removal) and as radar-relevant microwave absorption materials such as Ba-doped Mg–Zn ferrites.^4^

Among toxic metals, copper­(II) is particularly significant due to its dual role as an essential nutrient and a potential environmental toxin. While required in trace amounts, elevated Cu levels can provoke oxidative stress, gastrointestinal disturbances, and organ damage through mechanisms such as Fenton-like redox reactions.^5^ Because copper is widely used in agriculture and industrial processes, contamination of water sources and plant-based products (e.g., tea) poses a serious health concern.^6^

Regulatory agencies such as the World Health Organization (WHO) and the United States Environmental Protection Agency (US EPA) have set the maximum permissible concentration of copper in drinking water at 2.0 and 1.3 mg L^–^^1^, respectively.^7^ Direct measurement by flame atomic absorption spectrometry (FAAS), although accessible and cost-effective, often lacks sufficient sensitivity for such low-level detection. Therefore, incorporation of a selective and efficient preconcentration step is essential to enhance analytical performance.^8^

*d*-SPE coupled with FAAS offers a practical and powerful strategy for trace metal analysis, enabling high enrichment while maintaining procedural simplicity and low operational cost. In *d*-SPE, nanoscale sorbents are directly suspended in the sample, facilitating rapid analyte interaction and extraction. The process eliminates the need for traditional column materials and high solvent volumes, making it ideal for routine applications.^9^ When integrated with FAAS, *d*-SPE enhances sensitivity by concentrating the analyte and removing potential interferents in a single step. For example, Soylak and co-workers reported numerous nanosorbent-based *d*-SPE systems for Cu­(II) extraction, including magnetic graphene oxide modified with pyrocatechol violet, which yielded accurate and precise results in teas and water.^10^

Despite these advances, Ba-doped ZnFe_2_O_4_ spinel structures in *d*-SPE for Cu­(II) remain underexplored, particularly for direct FAAS analysis of food and environmental samples. While Ba–ZnFe_2_O_4_ nanohybrids have been synthesized for the adsorption of organic contaminants such as methylene blue,^4a^ there is no prior report on their application for heavy metal ion extraction or preconcentration. In contrast, other doped ZnFe_2_O_4_ systems—such as Mg-doped or TiO_2_-modified variants—have been more frequently studied for the removal of Pb^2^^+^, Cd^2^^+^, and Cu^2^^+^ from aqueous environments due to their enhanced surface area, altered charge distribution, or photocatalytic activity.^11^ However, Ba^2^^+^ incorporation may influence the spinel lattice by introducing relatively larger ionic radii. This could modify the surface basicity and thereby support stronger electrostatic or chelation-based interactions with Cu^2^^+^ ions. Ba-modified ZnFe_2_O_4_ could be an untapped, yet highly promising, material for the selective and efficient extraction of Cu­(II).

While various ferrite-based sorbents have been studied for heavy metal preconcentration, to the best of our knowledge, this is the first report specifically employing Ba-doped ZnFe_2_O_4_ in *d*-SPE–FAAS for Cu­(II) determination. We describe the preparation and structural characterization of this material in detail, followed by an optimized *d*-SPE–FAAS protocol that achieved a 62-fold LOD improvement factor, thereby providing a significant enhancement in the analytical response for Cu­(II) detection in tea infusions and environmental water matrices.

## 2 Experimental Section

### 2.1 Materials

All reagents employed in this study were of analytical grade and were used without further purification. Zinc sulfate heptahydrate (ZnSO_4_·7H_2_O), ferric chloride hexahydrate (FeCl_3_·6H_2_O), and barium chloride dihydrate (BaCl_2_·2H_2_O) were procured from Merck (Germany) and used as the starting materials in stoichiometric proportions. Nitric acid (HNO_3_, 65%) was added gradually to assist in homogenization and clarification of the reaction mixture. Sodium hydroxide (NaOH, 2 M) was used as a pH regulator to promote gel combustion and phase formation. All aqueous solutions were prepared using ultrapure deionized water.

Standard Cu­(II) solutions were prepared by serial dilution of a 1000 mg/L stock solution (ICP standard, High-Purity Standards, USA) by using nitric acid-stabilized ultrapure water. Buffer systems for pH optimization were prepared using potassium hydrogen phthalate (pH 3–6), tris­(hydroxymethyl)­aminomethane (TRIS; p*K*_a_ ≈ 8.1, effective range pH 7–9, used for pH 7–9), borax (pH 8–10), and disodium hydrogen phosphate (pH 11–12), all obtained from Merck.

### 2.2 Instrumentation

In this study, ultrapure water required for all solution preparations and dilutions was obtained using a Merck Millipore Direct-Q 3 UV purification system. Quantitative analysis of Cu­(II) ions was performed using a flame atomic absorption spectrometer (Thermo Scientific ICE-3000 series, USA). A copper hollow cathode lamp served as the radiation source, operating at a current of 4.0 mA and a spectral bandwidth of 0.5 nm. The instrument was set to measure the absorbance at 324.8 nm, the primary analytical wavelength for Cu­(II). An air–acetylene flame was employed, with acetylene supplied at a flow rate of 1.0 L min^−1^ and air as the oxidant adjusted to achieve a stoichiometric combustion environment. All measurements were carried out in triplicate to ensure analytical precision.

### 2.3 Characterizations

The structural and morphological properties of the synthesized Ba-doped ZnFe_2_O_4_ nanoparticles were characterized by using a range of instrumental techniques. X-ray diffraction (XRD) analysis was performed on a Rigaku Ultima IV diffractometer equipped with a Cu Kα radiation source (λ = 1.5406 Å), operating at 30 kV, across the 2θ range of 10–90°, confirming the spinel crystal structure. The surface morphology and particle size distribution were examined using field emission scanning electron microscopy (FESEM, Zeiss Sigma VP 300). Elemental composition and homogeneity of the particles were further analyzed via energy-dispersive X-ray spectroscopy (EDX) integrated with the FESEM system (Ametek EDAX). Fourier-transform infrared spectroscopy (FTIR) was employed to identify characteristic metal–oxygen stretching vibrations and confirm the formation of metal–oxide bonds within the spinel framework.

### 2.4 Synthesis of Barium-Doped ZnFe_2_O_4_ Nanoparticles

Various chemical synthesis protocols—such as sol–gel,^12^ hydrothermal,^13^ coprecipitation,^14^ and glycine-nitrate combustion methods^15^—have been employed to prepare spinel-type ferrite nanoparticles. The single-step autocombustion method stands out due to its operational simplicity, low temperature requirement, and the ability to yield highly homogeneous products. In this study, barium-doped zinc ferrite nanoparticles with the chemical formula Zn_0_._5__0_Ba_0_._5__0_Fe_2_O_4_ were successfully synthesized via the autocombustion technique.^16^

In this process, precursor salts including 0.5 g of ZnSO_4_·7H_2_O (approximately 1.74 mmol), 1.881 g of FeCl_3_·6H_2_O (6.96 mmol), and 0.425 g of BaCl_2_·2H_2_O (1.74 mmol) were weighed in stoichiometric ratios and dissolved in a defined volume of deionized water under continuous stirring. Ba doping level was fixed at *x* = 0.50, targeting the nominal composition Zn_0_._5__0_Ba_0_._5__0_Fe_2_O_4_. The precursor amounts were selected in stoichiometric proportions (Zn:Ba: Fe = 1:1:4), corresponding to 1.74 mmol of Zn^2^^+^, 1.74 mmol of Ba^2^^+^, and 6.96 mmol of Fe^3^^+^. Subsequently, 15 mL of diluted nitric acid (HNO_3_) was added dropwise to the solution to obtain a clear and homogeneous mixture. This acid treatment regulated pH and ensured complete dissolution.

The resulting homogeneous solution was heated on a magnetic hot plate at 100 °C for approximately 2 h to promote the gradual evaporation of the solvent. Over time, the solution transformed into a dark brown, viscous gel. This precursor gel was then subjected to thermal treatment at 200 °C for 15 min to initiate the self-sustained combustion reaction. During this process, rapid gas evolution was observed, which was attributed to the redox-activity generated by the organic components and nitrate ions. This led to the formation of a dry, carbon-depleted, and shrunken organic matrix.

Upon partial pyrolysis and combustion, a brownish solid residue was obtained, composed predominantly of metal-oxide-based ash containing ZnFe_2_O_4_ nanocrystals. This solid product was then ground manually using an agate mortar and pestle to yield a fine, homogeneous powder.

### 2.5 Extraction Procedure

The preconcentration of Cu­(II) ions was performed using the *d*-SPE-FAAS protocol optimized for spinel-type Ba-doped ZnFe_2_O_4_ nanoparticles. A certified 1000 mg·L^–^^1^ Cu­(II) stock solution (High-Purity Standards, USA) was serially diluted to prepare calibration standards within the linear range. For each extraction experiment, 40 mL of either standard or real sample solution was used, and the pH was adjusted to 9.0 by directly adding 1 mL of pH 9.0 buffer solution. Subsequently, 40 mg of Ba-doped ZnFe_2_O_4_ nanopowder was added, and the mixture was vortexed for 60 s to ensure adequate dispersion and interaction for efficient adsorption.

Following the adsorption step, the suspension was centrifuged at 8000 rpm to separate the solid and liquid phases, and the supernatant was discarded. The Cu­(II)-loaded solid phase was then treated with 0.3 mL of concentrated HNO_3_ and vortexed for 60 s to desorb the retained metal ions. The eluate was separated by a second centrifugation and collected for FAAS analysis. After desorption with 0.3 mL of concentrated HNO_3_ (≈15.8 M), the eluate was quantitatively diluted to a final volume of 3.0 mL with deionized water and introduced to FAAS, corresponding to approximately 1.6 M HNO_3_ in the nebulized solution. Between runs, the sample introduction system was rinsed (DI → 1% v/v HNO_3_ → DI). Importantly, several reports in the analytical chemistry literature have also demonstrated the safe introduction of eluates containing 1–3 M HNO_3_ into FAAS systems without adverse effects, provided that calibration solutions are matrix-matched and appropriate rinse protocols are applied.^17^

All absorbance measurements were blank-corrected. Procedural blanks, prepared by using ultrapure water and subjected to the same extraction and elution steps, consistently yielded no detectable Cu signal. These blank values were subtracted from sample signals before calibration and quantification to eliminate potential background contributions.

To verify that Cu­(II) remained in the dissolved fraction at the working pH, an additional control experiment was performed before the sorbent addition. A 250 ng mL^–^^1^ Cu­(II) standard was prepared at pH 9.0 using TRIS buffer. The solution was passed through a 0.22 μm PTFE membrane filter and analyzed by FAAS together with an unfiltered aliquot under the same conditions. No statistically significant difference was observed between filtered and unfiltered samples (*n* = 3, *t* test, *p* > 0.05), confirming that Cu­(II) remained in the dissolved phase under the employed alkaline conditions. This control ensured that subsequent extraction was based on the adsorption of dissolved species rather than the capture of colloidal precipitates.

All extraction parameters—including pH, sorbent amount, eluent volume, mixing time, and mixing method (vortex mixing during adsorption and ultrasonic mixing during desorption stages)—were systematically evaluated through univariate optimization experiments. The optimum values obtained for each variable were then used to assess the method’s analytical performance.

Method performance characteristics were determined in terms of the limit of detection (LOD), limit of quantification (LOQ), linear dynamic range, correlation coefficient (*R* ^2^), and precision (%RSD). The LOD and LOQ values were calculated using the slope (*S*) of the calibration curve and the standard deviation (SD) of seven replicate measurements at the lowest concentration level according to the following equations:

LOD=3.3×SDS 1

LOQ=10×SDS 2

Additionally, the LOD improvement factor was calculated to quantify the signal enhancement provided by the preconcentration step. LOD improvement factor was defined as the ratio of the detection limit obtained by direct FAAS (LOD_FAAS_) to the detection limit achieved after applying the *d*-SPE–FAAS method (LOD_*d*‑SPE_):

$$\mathrm{LOD}\;\mathrm{improvement}\;\mathrm{factor}\;=\frac{{\mathrm{LOD}}_{\mathrm{FAAS}}}{{\mathrm{LOD}}_{d‐\mathrm{SPE}-\mathrm{FAAS}}}$$ 3

To further confirm the stability of the sorbent under the applied conditions, leaching tests for Ba, Zn, and Fe were performed at pH 9.0 with a nitric acid elution. No measurable release was detected by FAAS, indicating that the sorbent remained stable during extraction and did not contribute to interfering ions.

In addition, batch adsorption experiments were conducted at pH 9.0 to evaluate the intrinsic sorption capacity of Ba-doped ZnFe_2_O_4_ nanoparticles. For each experiment, 50 mg of sorbent was equilibrated with 40 mL of Cu­(II) solutions with initial concentrations ranging from 10 to 200 mg L^−1^. The suspensions were agitated on a rotary shaker for 60 min to ensure equilibrium was reached, followed by centrifugation and collection of the supernatant. After separation, residual Cu­(II) was measured by FAAS, and *q*_*e*_ values were calculated. The data were fitted to the Langmuir model to estimate *q*_max_.

### 2.6 Preparation of the Real Samples

To evaluate the applicability of the developed *d*-SPE–FAAS method to real-world matrices, green tea infusion, tap water, and domestic wastewater samples were analyzed. The green tea infusion was prepared by steeping 2 g of commercially available tea in 100 mL of boiling ultrapure water for 10 min. After being cooled to room temperature, the infusion was filtered through a 0.45 μm membrane and subsequently diluted 20-fold with ultrapure water to minimize matrix effects. Tap water was collected from the laboratory at Van Yüzüncü Yıl University using acid-washed polyethylene containers, filtered through a 0.45 μm membrane, and diluted 20-fold before analysis. The domestic wastewater sample was obtained from the influent stream of the VASKİ municipal treatment facility. After the settling of large particulates, the supernatant was filtered and diluted 50-fold with ultrapure water to reduce potential interferences arising from organic or inorganic matrix constituents.

## 3 Results and Discussions

### 3.1 Characterizations of Ba-Doped ZnFe_2_O_4_ Nanoparticles

The morphological and elemental characteristics of the synthesized Ba-doped ZnFe_2_O_4_ nanoparticles were investigated by SEM, EDX, FTIR, and XRD analyses.

SEM analysis revealed that the synthesized nanomaterials possess a rough, irregular, and highly agglomerated morphology, as shown in Figure 1a,b. The image taken at higher magnification (Figure 1b) highlights the formation of spherical-like aggregates composed of interconnected primary nanoparticles. This structural feature suggests an increased surface area and porosity, which are highly favorable for adsorption-based applications. Similar microstructural arrangements have been reported in the literature for doped ferrites synthesized via autocombustion methods, indicating the suitability of the approach used in this study.^18^

**Figure 1:**
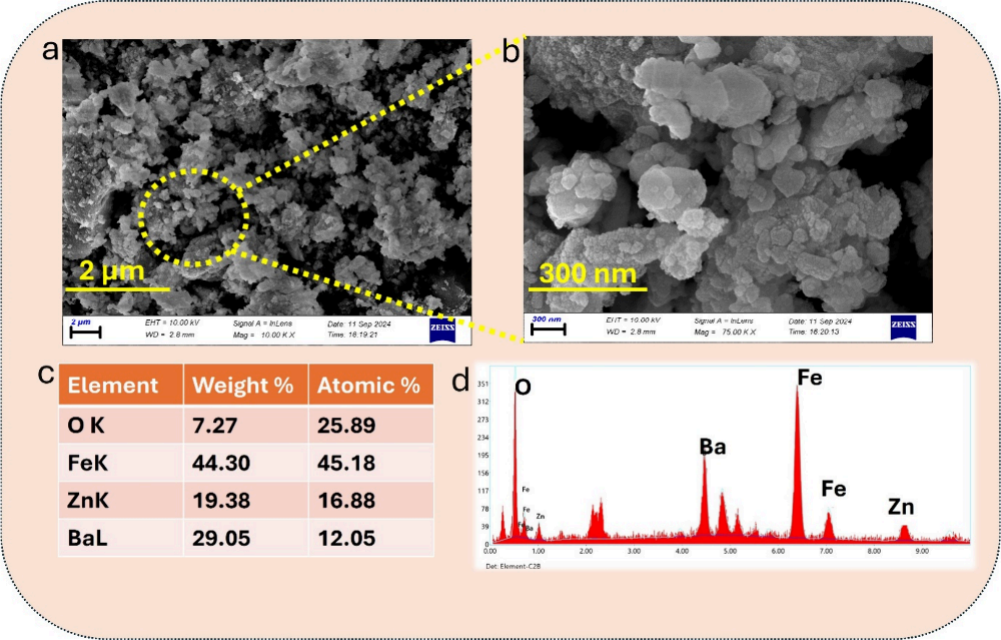
(a, b) SEM images of Ba-doped ZnFe_2_O_4_ nanoparticles. (c) EDX elemental composition of Fe, Zn, Ba, and O. (d) EDX spectrum of the nanoparticles.

EDX spectroscopy (Figure 1c,d) confirmed the successful elemental composition of the nanomaterials. The elemental spectrum displayed characteristic peaks for Zn, Fe, O, and Ba, indicating successful doping of Ba^2^^+^ into the ZnFe_2_O_4_ spinel lattice. The atomic percentages were calculated as Fe (45.18%), Zn (16.88%), O (25.89%), and Ba (12.05%). Notably, the incorporation of Ba, a relatively larger ion (ionic radius: 1.35 Å),^19^ into the spinel structure without forming additional phases suggests lattice accommodation, likely at tetrahedral or octahedral sites, consistent with prior studies.^20^

FTIR analysis (Figure 2a) further validated the spinel structure. The spectrum displayed prominent absorption bands around 684, 580, and 534 cm^–^^1^, which are characteristic of metal–oxygen stretching vibrations in the tetrahedral and octahedral sites of spinel ferrites.^21^ These bands are attributed to Fe–O and Zn–O vibrations within the spinel framework. The small shifts observed in peak positions compared to pure ZnFe_2_O_4_ may be attributed to lattice distortion due to Ba substitution. This behavior aligns with previous reports on rare-earth-doped ferrites.^22^

**Figure 2:**
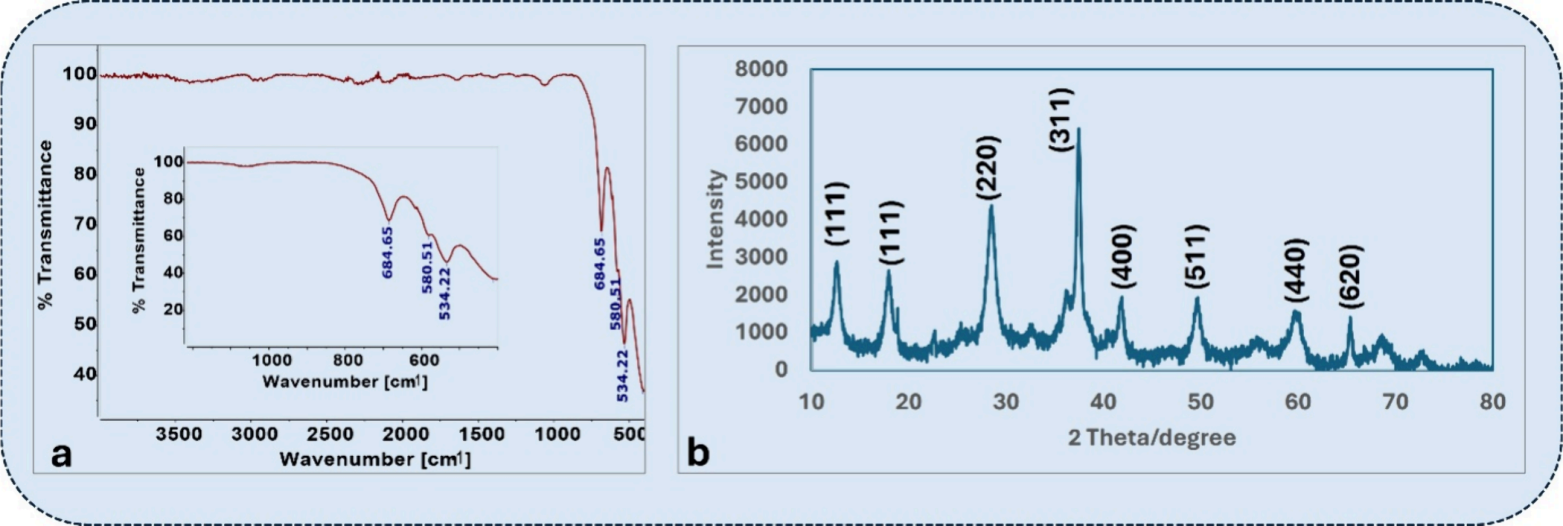
(a) FTIR spectrum of Ba-doped ZnFe_2_O_4_ nanoparticles, displaying characteristic metal–oxygen vibrational bands. (b) XRD pattern confirming the formation of a single-phase spinel ferrite structure, consistent with the standard JCPDS card no. 22–1012.

XRD analysis (Figure 2b) confirmed the single-phase spinel crystallographic structure of the material. The diffraction peaks at 2θ ≈ 30.2, 35.5, 43.2, 53.4, 57.2, and 62.8° correspond to the (220), (311), (400), (422), (511), and (440) planes, respectively, which are well indexed to the standard spinel structure of ZnFe_2_O_4_ (JCPDS No. 22–1012). The absence of impurity peaks indicates the phase purity of the synthesized material. Moreover, no secondary peaks corresponding to BaO or BaFe_2_O_4_ were observed, suggesting that Ba^2^^+^ was effectively doped into the spinel matrix without forming separate crystalline phases. This result is consistent with earlier reports demonstrating that Ba^2^^+^ doping up to similar levels can be structurally integrated into spinel ferrite lattices without phase segregation, preserving the single-phase spinel structure.^20,23^

In summary, SEM and EDX results confirmed the nanostructured morphology and elemental composition of Ba-doped ZnFe_2_O_4_, while FTIR and XRD analyses verified the successful incorporation of Ba into the spinel lattice without altering its crystal phase. These findings suggest that the synthesized nanoparticles are structurally suitable and chemically robust for their intended application in trace-level Cu­(II) adsorption and recovery.

### 3.2 Analytical Method Optimization

To achieve reliable and reproducible Cu­(II) quantification, critical experimental parameters affecting the extraction efficiency of the developed *d*-SPE–FAAS method were systematically optimized. These parameters include solution pH, sorbent dosage, eluent volume, mixing method, and time, all of which play a vital role in the adsorption–desorption performance. Each variable was evaluated through univariate experiments, and the optimized conditions were subsequently used for the analytical performance validation.

#### 3.2.1 Optimization of pH

The pH of the aqueous phase critically influences the adsorption process by modulating both the surface charge of the sorbent and the chemical speciation of metal ions such as Cu­(II). In this study, the effect of pH on the extraction efficiency of Cu­(II) was systematically examined over the range of 2.0 to 10.0 using 40 mL of a 250 ng mL^–1^ Cu­(II) solution buffered with 1 mL of an appropriate buffer. All experiments were conducted using 30 mg of Ba-doped ZnFe_2_O_4_ nanoparticles and a constant vortex mixing time of 30 s for both adsorption and desorption steps.

As shown in Figure 4A, Cu­(II) recovery was negligible under strongly acidic conditions (pH 2–6), primarily due to the excessive protonation of surface functional groups on the nanosorbent, which hinders coordination with metal ions. Furthermore, competition between H^+^ and Cu^2^^+^ ions for active adsorption sites significantly reduces uptake efficiency in this pH range.^24^

A sharp increase in absorbance was observed starting at pH 7.0, with a maximum extraction efficiency recorded at pH 9.0. This enhancement is attributed to the effective deprotonation of surface hydroxyl or carboxyl groups, which promotes the formation of surface–metal complexes via electrostatic attraction and inner-sphere complexation.^25^ At higher pH values, the nanoparticle surface likely becomes more negatively charged, facilitating stronger interactions with divalent Cu­(II) cations.

Notably, under pH 9.0 conditions, the signal was calculated as 11.4, obtained by taking the ratio of the FAAS absorbance measured after extraction to the direct FAAS absorbance of Cu­(II) at 250 ng mL^–^^1^. Consequently, pH 9.0 was selected as the optimum value for all subsequent extractions, offering a well-balanced compromise among maximum recovery, minimal matrix interference, and high repeatability.

A frequently raised concern in analytical chemistry is the potential precipitation of Cu­(II) as Cu­(OH)_2_ at alkaline pH values, particularly above pH 8.5. To address this, several factors were carefully controlled in the present study. First, before the addition of the sorbent, the Cu­(II) solution adjusted to pH 9.0 (buffered with 1.0 mL of 0.10 M TRIS stock per 40 mL sample; final ionic strength ≈ 2.5 mM) was subjected to verification by 0.22 μm filtration followed by FAAS analysis. No significant loss of Cu was detected after filtration compared to the unfiltered aliquots (*n* = 3), confirming that Cu remained in the dissolved fraction under these conditions.

Additionally, the working concentration of Cu­(II) (≤300 ng mL^–^^1^) is far below the solubility threshold of Cu­(OH)_2_ (Ksp = 2.2 × 10^–^^20^),^26^ and the use of TRIS buffer, known to form weak Cu–TRIS complexes,^27^ further stabilizes Cu­(II) in solution and reduces the likelihood of precipitation. Moreover, the nanosorbent surface contains active coordination sites (e.g., Fe–O^–^, Zn–O^–^, Ba–O^–^) that strongly bind Cu^2^^+^, promoting adsorption over hydrolytic precipitation.^28^ Collectively, these results support the interpretation that Cu­(II) remained in a soluble and adsorbable state at pH 9.0 and that the extraction process relied on surface complexation rather than capture of colloidal precipitates.

Reported pHpzc values for ZnFe_2_O_4_-based spinels and their composites vary within the range of ∼4.3–7.5, depending on composition and synthesis route. For example, a ZnFe_2_O_4_/activated carbon nanocomposite (ZFAC) exhibited a pHpzc of 4.3, reflecting strong surface acidity.^29^ Similarly, Mg-doped ZnFe_2_O_4_ showed a slightly higher pHpzc of 5.1, consistent with the modifying effect of Mg^2^^+^ substitution.^30^ Spherical ZnFe_2_O_4_ nanoparticles (ZF-NPS) were reported with a pHpzc of 5.8, indicating that under near-neutral to alkaline conditions, the surface becomes negatively charged.^3^ A ZnFe_2_O_4_/SnO_2_ composite demonstrated a somewhat higher value of 6.75, reflecting the contribution of SnO_2_ to the surface chemistry.^31^ Finally, ZnFe_2_O_4_ investigated for malachite green adsorption exhibited a pHpzc of 7.5, the upper range reported for this spinel family.^32^

Taken together, these reports clearly indicate that the pHpzc of ZnFe_2_O_4_-based materials is consistently below our working pH of 9.0. Under such conditions, the nanosorbent surface is expected to be negatively charged due to deprotonation of surface M–OH groups (M = Fe, Zn, Ba). This electrostatic environment strongly favors the attraction and complexation of divalent Cu­(II) cations. Notably, the postadsorption SEM–EDX spectrum and elemental mapping (Figure 3) obtained in this study confirmed the presence and homogeneous distribution of Cu on the nanosorbent surface. The observation of Cu coexisting with the spinel lattice elements (Fe, Zn, and Ba) indicates that the uptake mechanism is largely consistent with deprotonation-assisted surface complexation.

**Figure 3:**
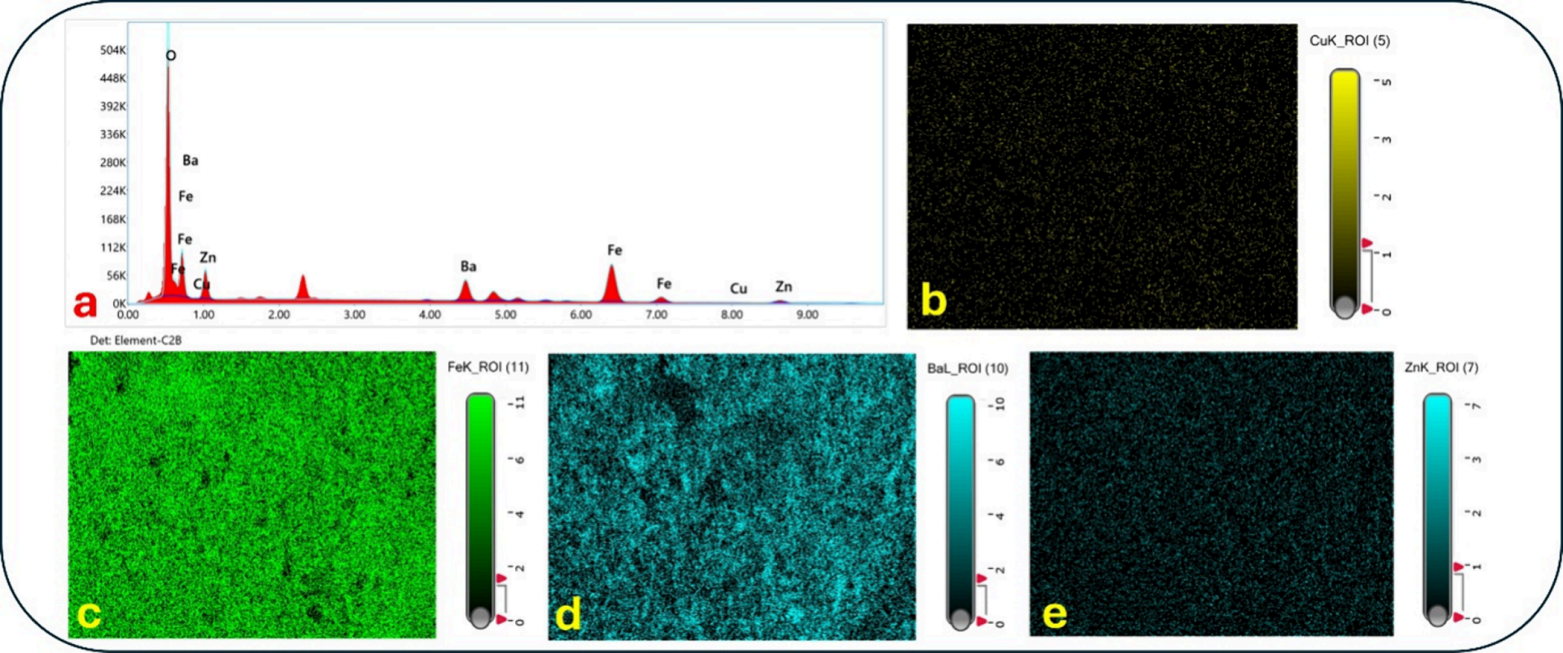
Postadsorption SEM–EDX analysis of Ba-doped ZnFe_2_O_4_: (a) EDX spectrum showing characteristic peaks of Ba, Fe, Zn, O, and Cu; (b) Cu elemental mapping confirming surface localization of adsorbed Cu­(II); (c) Fe mapping; (d) Ba mapping; (e) Zn mapping. The data collectively indicate that Cu­(II) uptake occurs via surface adsorption rather than bulk precipitation.

In addition, the volume of the buffer solution used to adjust the sample pH was also optimized (see Figure 4F). It was found that increasing the buffer volume from 0.5 to 1.0 mL significantly improved the stability and reproducibility of solution pH during extraction. Beyond 1.0 mL, no further enhancement in extraction efficiency was observed. Therefore, 1 mL of buffer was used in all experiments to ensure consistent pH control without excessive sample dilution.

**Figure 4:**
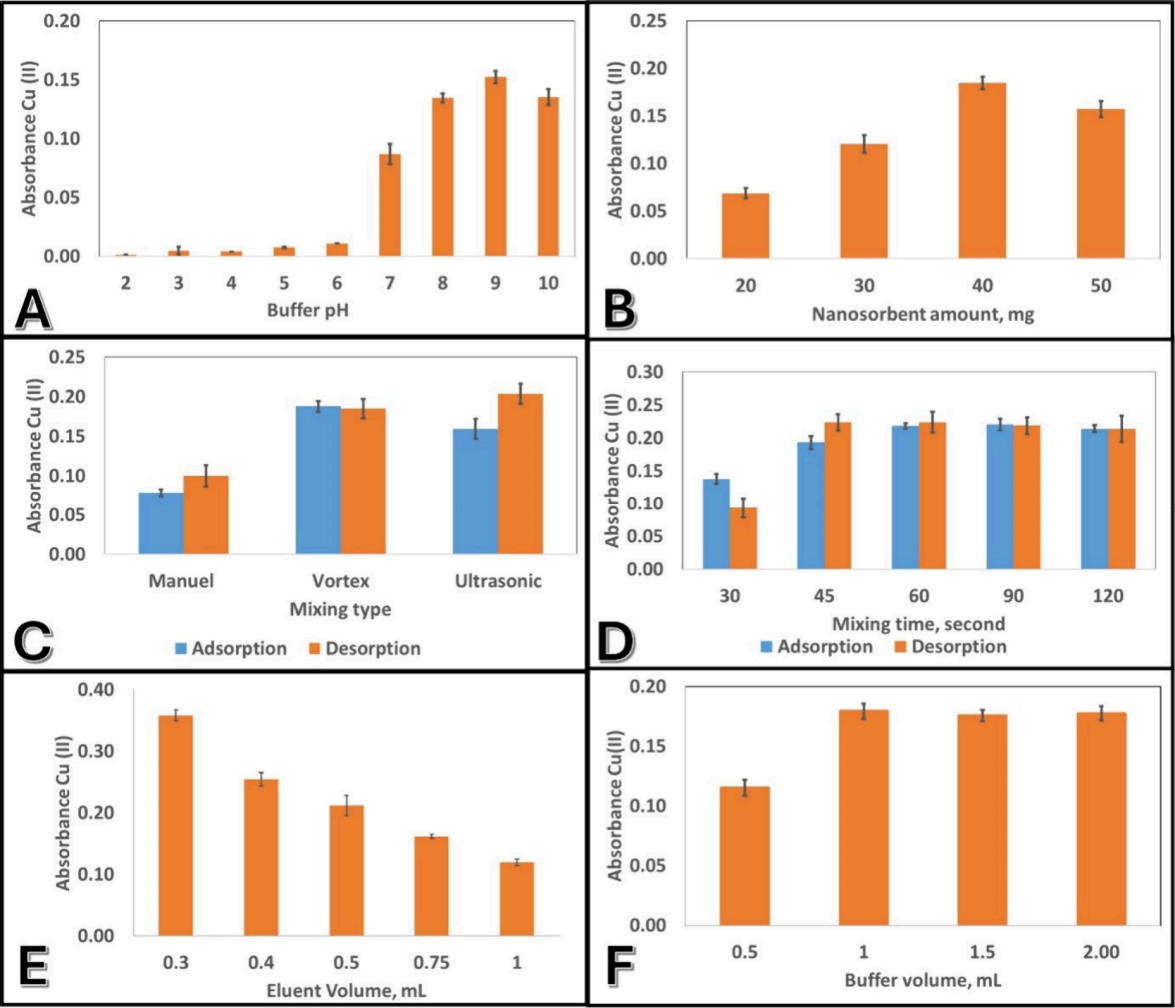
Effect of various experimental parameters on the *d*-SPE-FAAS: (A) pH (2–10; 30 mg Ba-doped ZnFe_2_O_4_, 1 mL buffer, vortex 30 s adsorption and desorption, 0.5 mL HNO_3_ desorption),(B) nanosorbent amount (20–50 mg; pH 9.0, vortex 30 s adsorption, ultrasonic 30 s desorption, 0.5 mL HNO_3_),(C) mixing type (manual, vortex, ultrasonic; pH 9.0, 40 mg sorbent, 0.5 mL HNO_3_),(D) mixing time (30–120 s; same conditions as C), (E) eluent volume (0.3–1.0 mL HNO_3_; pH 9.0, 40 mg sorbent), (F) buffer volume (0.5–2.0 mL; pH 9.0, 40 mg sorbent, vortex 30 s adsorption and desorption, 0.5 mL HNO_3_ desorption). All experiments were carried out with 40 mL of 250 ng mL^–^^1^ Cu­(II) solution using Ba-doped ZnFe_2_O_4_ nanoparticles and concentrated (65%) HNO_3_ as the eluent.

#### 3.2.2 Optimization of the Amount of Nanosorbent

The amount of nanosorbent is a critical factor that directly affects the number of available active sites for metal ion binding during the extraction process. To determine the optimal quantity of Ba-doped ZnFe_2_O_4_ nanoparticles, batch experiments were performed by varying the adsorbent mass from 20 to 50 mg under previously optimized conditions: pH 9.0, 40 mL sample volume (250 ng mL^–1^ Cu­(II)), vortex agitation for 30 s (both adsorption and desorption), and 0.5 mL of HNO_3_ as the eluent.

As illustrated in Figure 4B, the Cu­(II) absorbance signal increased steadily from 20 to 40 mg of nanosorbent, indicating improved retention due to the availability of more active sites such as Fe–O^–^, Zn–O^–^, and Ba–O^–^ surface groups. The maximum signal was obtained at 40 mg, beyond which a decline was observed at 50 mg. This decrease may be attributed to several factors: (i) excessive sorbent can lead to nanoparticle aggregation, effectively reducing the accessible surface area and blocking adsorption sites; (ii) higher solid content may hinder efficient dispersion and interaction with the analyte due to mass transfer limitations; and (iii) overloading of sorbent could retain eluent during desorption, reducing recovery efficiency. Thus, 40 mg was selected as the optimal nanosorbent quantity for subsequent extractions, providing a balance between maximum uptake, reproducibility, and efficient elution.

#### 3.2.3 Optimization of Mixing Type and Time

The efficiency of the solid-phase extraction process is significantly influenced by the mixing mechanism, which governs the interaction between the adsorbent and target analyte. Proper mixing facilitates diffusion in solution during both adsorption and desorption stages, thereby accelerating mass transfer and maximizing the interaction.

In this study, the effect of three different mixing methods—manual, vortex, and ultrasonic—on Cu­(II) adsorption and desorption efficiency was investigated (Figure 4C). Manual mixing, due to its limited mechanical energy, resulted in inadequate mass transfer and, consequently, lower adsorption/desorption efficiency. Vortex mixing notably enhanced the adsorption step, likely due to improved dispersion of nanoscale particles and more effective exposure of active surface areas. The highest desorption efficiency was achieved with ultrasonic mixing, which is consistent with literature reports indicating that ultrasonic waves generate microcavitation in the solution, promoting the release of metal ions from the sorbent surface.^33^

The effect of mixing time was also evaluated between 30 and 120 s, and the results are presented in Figure 4D. At 60 s of mixing, both adsorption and desorption efficiencies reached a plateau, beyond which improvements were negligible. The absorbance signal obtained at 60 s was approximately 1.58 times higher than that observed at 30 s. This indicates that kinetic equilibrium was achieved and the active sites on the sorbent surface reached saturation.

These optimization studies were conducted using a 250 ng mL^–1^ Cu­(II) standard solution at pH 9.0, with 0.5 mL of HNO_3_ as the eluent and 40 mg of nanosorbent.

In conclusion, vortex mixing was found to be optimal for the adsorption step, while ultrasonic mixing was optimal for the desorption step. A mixing duration of 60 s was established as ideal for both processes. These conditions not only provide high efficiency but also reduce the operational time, making the method suitable for practical applications.

#### 3.2.4 Optimization of Eluent Volume

In *d*-SPE-FAAS systems, the volume of the eluent used during the desorption phase is a critical parameter, directly influencing the recovery of the adsorbed analyte. An insufficient eluent volume may lead to incomplete desorption, whereas excessive volumes can cause unnecessary dilution, reducing sensitivity and the LOD improvement factor.

In this study, concentrated HNO_3_ was used as the eluent to ensure the rapid and efficient desorption of Cu­(II) ions from the nanosorbent surface. Notably, only small volumes (0.3–1.0 mL) of eluate were used, and after appropriate dilution, no clogging, corrosion, or signal instability was observed in the FAAS system. Moreover, nitric acid is among the most commonly used eluents in trace metal determinations due to its strong protonation ability and compatibility with FAAS.^34^

The influence of eluent volume on the analytical signal was evaluated over the range of 0.3 to 1.0 mL (see Figure 4E). The highest absorbance signal was recorded at 0.3 mL, after which a steady decrease was observed with an increasing volume. This behavior can be attributed to the dilution effects. Although the total amount of desorbed Cu­(II) may remain constant, its concentration in the eluent decreases with increasing volume, resulting in lower absorbance values. At 1.0 mL, the signal dropped by more than 60% relative to the 0.3 mL condition. These findings confirm that 0.3 mL of concentrated HNO_3_ is sufficient for quantitative desorption and optimal for achieving high sensitivity.

Specifically, after desorption with 0.3 mL of concentrated HNO_3_ (≈15.8 M), the eluate was quantitatively diluted to a final volume of 3.0 mL with deionized water before nebulization, yielding a final acidity of ∼1.6 M HNO_3_.

In conclusion, 0.3 mL of concentrated nitric acid, subsequently diluted to 3.0 mL before the FAAS measurement, was selected as the optimal eluent volume, ensuring complete desorption, maximum sensitivity, and system compatibility.

### 3.3 Analytical Figures of Merit

Before validation, the extraction conditions were optimized to ensure a maximum analytical performance. The final selected parameters were as follows: solution pH of 9.0 (adjusted with 1.0 mL buffer), nanosorbent dosage of 40 mg, eluent type of concentrated HNO_3_ with an optimized volume of 0.3 mL, and mixing conditions consisting of vortex agitation for adsorption and ultrasonic treatment for desorption, each applied for 60 s. A sample volume of 40 mL was used in all of the experiments. These optimized conditions provided the best compromise between high extraction efficiency, reproducibility, and sensitivity and were employed for all subsequent validation studies.

The analytical performance of the developed *d*-SPE-FAAS method for Cu­(II) determination was evaluated in terms of key validation parameters, including linearity, limit of detection (LOD), and correlation coefficient (*R* ^2^). The LOD, LOQ, and linearity values reported in this work were calculated from aqueous calibration standards. For real sample analyses, matrix-matched calibration curves were employed to account for possible matrix effects.

Additionally, leaching experiments were carried out for Ba, Zn, and Fe under the working conditions (pH 9.0 and nitric acid elution). No measurable release was detected by FAAS (all values below the instrumental LOD, < 0.05 mg L^–^^1^), confirming that Ba^2^^+^ release was negligible. These results ensure that Cu­(II) uptake was not biased by lattice cation leaching and that the proposed method is both reliable and safe for analytical applications.

The calibration curve for Cu­(II) exhibited excellent linearity in the concentration range of 5.0–300 ng mL^–^^1^, with a correlation coefficient (*R* ^2^) of 0.9992, indicating a highly reliable linear response. The LOD (limit of detection) and LOQ (limit of quantification) were determined to be 0.67 and 2.23 ng mL^–^^1^, respectively. The method also exhibited acceptable precision, with a relative standard deviation (RSD) of 5.5% (10 ng mL^–^^1^, *n* = 7), a high LOD improvement factor of 62, a preconcentration factor (PF) of 30 (calculated according to the *C*_final_/*C*_initial_ approach), and an enhancement factor (EF) of 29 (based on calibration slope ratios). This low detection limit reflects the high sensitivity of the method and its suitability for trace-level monitoring of Cu­(II) in environmental and food samples.

These results demonstrate that the developed method provides a wide dynamic range, strong linearity, and excellent sensitivity, making it a robust analytical tool for trace Cu­(II) determination. Also, a comparative summary of the proposed method and similar studies reported in the literature is provided in Table 1.

**Table 1:** Comparison of the Proposed *d*-SPE-FAAS Method with Literature-Reported Approaches

method/adsorbent for Cu(II) determination	LOD^a^/LOQ^b^ (ng mL^–1^)	linear range (ng mL^–1^)	EF	real sample	ref
*d*-SPE-FAAS/Ba-doped ZnFe_2_O_4_	0.67/2.23	5.0–300	29	green tea, wastewater, tap water	this study
SA-*d-*SPE/dithizone@PAA	0.06/0.2	0.20–125.00	50	fortified vegetables and barbecue samples	^35^
M-D-μSPE^c^/Fe–Ni@ACC^d^ nanocomposite	0.69/2.29	–	40	tap water, cigarette, human hair, and black tea samples	^36^
MDMSPE^e^-ICP-OES/MCOF-DES^f^	0.16/0.54	0.4–700	30	medicinal plants and environmental samples	^37^
column-SPE-ICP-OES/MWCNTs-CO-Sac^g^	0.09/–	–	75	water and soil samples	^38^
SPE-FAAS/PS–PPDOT^h^	0.56/2.0	3.0–120	41	water, soil, and food samples	^39^
MSPE-FAAS/MMSM-PEI[Table-fn t1fn9]	140/460	–	–	preserved eggs	^40^
EDXRF^j^/CoFe_2_O_4_	32/107	107–1000	–	sugar cane spirit	^41^
MSPE-FAAS NiFe_2_O_4_	2.1/7.0	7.0–2000	30	water and food samples	^42^
FAASMnFe_2_O_4_ @ alunite	0.91/–	4.0–150	80	food, water	^43^

a Limit of detection.

b Limit of quantification.

c Dispersive micro solid-phase extraction.

d Activated carbon cloth.

e Magnetic dispersive micro solid-phase extraction.

f Magnetic covalent organic framework (MCOF) modified by a new deep eutectic solvent.

g Multiwalled carbon nanotubes-COOH with saccharine.

h Polystyrene modified by 1-phenyl-1,2-propanedione-2-oxime thiosemicarbazone.

j Magnetic mesoporous silica microsphere.

k Energy-dispersive X-ray fluorescence spectrometry, -: not reported in the corresponding study.

The precision results summarized in Table 2 demonstrate that the proposed *d*-SPE–FAAS method provides acceptable repeatability and intermediate precision across the tested concentration levels. At 20 ng mL^–^^1^, intraday and interday %RSD values were 5.3 and 7.3%, while at 50 ng mL^–^^1^, they were 5.9 and 6.2%, respectively. These values are consistent with typical performance criteria for trace metal determination, indicating that the method maintains a stable response both within a single analytical session and across multiple days.

**Table 2:** Intraday Repeatability and Interday Intermediate Precision of the Proposed *d*-SPE–FAAS Method for Cu­(II) Determination^a^

level (ng mL^–^^1^)	mean_found_ (ng mL^–^^1^)	RSD_intra_ (%)	mean_all (ng mL^–^^1^)	RSD_inter_ (%)
20	19.1	5.3	19.0	7.3
50	48.4	5.9	48.1	6.2

a In both table entries, intraday *n* = 6 and interday *n* = 3 × 6 = 18.

Beyond analytical validation, a simple adsorption isotherm study was conducted to evaluate the sorbent’s intrinsic performance. Equilibrium data fitted well to the Langmuir model (*R* ^2^ > 0.99), and the calculated maximum monolayer adsorption capacity (*q*_m__a__*x*_) was 104.2 mg g^–^^1^ for Cu­(II). This capacity is comparable to that of an activated carbon/NiFe_2_O_4_ magnetic composite (*q*_m__a__*x*_ = 105.8 mg g^–^^1^).^44^

### 3.4 Real Sample Application

To evaluate the applicability and reliability of the proposed *d*-SPE-FAAS method in complex matrices, recovery studies were conducted using real samples including tap water, wastewater, and green tea infusions. These matrices were selected to represent a wide range of environmental and beverage-related sample types, each posing distinct analytical challenges due to their unique compositions.

Tap water and wastewater are commonly tested environmental matrices in trace metal analysis. While tap water generally presents a relatively clean matrix with low organic and particulate content, wastewater is a complex medium containing high concentrations of organic matter, suspended solids, and various interfering ions, such as Ca^2^^+^, Mg^2^^+^, Na^+^, and Cl^–^, which can potentially affect extraction and detection efficiency.^24a,45^

Nanomaterials, in addition to their analytical use in food samples, are increasingly employed in the food industry to enhance product quality, stability, and functionality.^46^ Green tea, on the other hand, is a beverage matrix rich in polyphenols, tannins, and caffeine, which may interact with metal ions and sorbent surfaces, potentially hindering quantitative recovery.^24a,47^

To further minimize matrix effects and ensure the accuracy of the quantification, matrix-matched calibration curves were employed for each sample type. Calibration was performed by spiking real samples at three concentration levels (20, 50, and 100 ng mL^–^^1^), and the resulting standard additions were used to construct the calibration curves. This approach allowed the calibration standards to better reflect the chemical matrix of the real samples, accounting for potential signal suppression or enhancement caused by the matrix components.

The Cu­(II) recoveries from the studied real samples ranged between 94.2 and 105.6%, indicating excellent reliability and minimal matrix interference. To ensure accurate quantification, all real samples were first pretreated through 0.45 μm membrane filtration to remove particulates and then diluted with ultrapure water (20-fold for green tea and tap water; 50-fold for wastewater) to mitigate matrix-related interferences before analysis. Under these conditions, the native Cu concentrations were determined as 27.8 ± 2.3 in tap water, 155.9 ± 7.2 ng mL^–1^ in green tea, and 2338.5 ± 128.2 ng mL^–1^ in wastewater (Table 3). Spike–recovery experiments at three concentration levels (20, 50, and 100 ng mL^–1^, added on the original scale) were conducted by using matrix-matched calibration curves, prepared under identical dilution and buffer conditions.

**Table 3:** Recovery Results with Their Standard Deviations for Cu­(II) in Wastewater, Green Tea, and Tap Water Matrices (*N* = 3)^a^

real sample	native Cu(II) (ng mL^–1^)^b^	Cu(II) spiked concentration (ng mL^–1^)	recovery (%)^b^
wastewater	2338.5 ± 128.2	20	97.2 ± 7.2
		50	96.6 ± 9.0
		100	96.4 ± 8.3
green tea	155.9 ± 7.2	20	94.2 ± 6.8
		50	100.4 ± 6.3
		100	101.7 ± 7.7
tap water	27.8 ± 2.3	20	98.3 ± 7.0
		50	103.7 ± 4.8
		100	105.6 ± 8.6

a Spike recoveries were obtained using matrix-matched calibration curves prepared under the same dilution and buffer conditions as the analyzed matrices.

b Data are presented as mean ± SD.

### 3.5 Effect of Interfering Ions

To evaluate the selectivity of the developed *d*-SPE-FAAS method for Cu­(II) determination, systematic interference studies were conducted. These involved introducing various commonly coexisting ions into Cu­(II)-containing solutions to simulate the composition of environmental and food matrices, which are typically complex due to the presence of a variety of dissolved inorganic species (Table 4).

**Table 4:** Effect of Potentially Interfering Ions on Cu­(II) Recovery under Optimized *d*-SPE–FAAS Conditions (*N* = 3)

ion	added salt	concentration (mg L^–1^)	recovery (%)
Co^2+^	CoCl_2_	2.0	93.4 ± 6.2
Ni^2+^	Ni(NO_3_)_2_	2.0	91.3 ± 7.8
Cr^3+^	Cr(NO_3_)_3_	2.0	90.2 ± 7.1
Pb^2^^+^	Pb(NO_3_)_2_	2.0	88.8 ± 5.7
Cd^2^^+^	CdCl_2_·H_2_O	2.0	83.5 ± 6.4
Fe^3^^+^	Fe(NO_3_)_3_	2.0	90.1 ± 5.1
Zn^2^^+^	Zn(NO_3_)_2_	2.0	89.2 ± 6.9
Ba^2^^+^	Ba(NO_3_)_2_	2.0	87.5 ± 7.1
Mg^2+^	MgCl_2_	50	92.1 ± 5.7
Cl^–^, Ca^2+^	CaCl_2_	50	94.3 ± 7.1
NO_3_^–^, K^+^	KNO_3_	50	94.4 ± 4.3
Na^+^, Cl^–^	NaCl	50	91.8 ± 5.1
SO_4_^2–^	Na_2_SO_4_	50	93.2 ± 4.7

The tested cations included transition metals (Co^2+^, Ni^2^^+^, and Cr^3^^+^), which are known to form stable complexes with chelating agents, and alkaline/alkaline earth metals (Mg^2^^+^, Ca^2^^+^, K^+^, and Na^+^), which often exist in environmental waters at high concentrations. Common anions (Cl^–^, NO_3_^–^, and SO_4_^2^^–^) were also included to assess the impact of ionic strength and potential ionic competition on Cu­(II) binding. Each potential interferent was added at a significantly higher concentration relative to Cu­(II), mimicking realistic and challenging sample conditions. For instance, interfering transition metals were spiked at 2.0 mg L^–^^1^, while alkali/alkaline earth metals and anions were added at 50 mg L^–^^1^. Despite this, Cu­(II) recoveries consistently ranged from 83.5 to 94.4%, with acceptable standard deviations (±4.3 to ±7.8), indicating that the extraction efficiency was not significantly compromised.

In addition, the most mechanistically relevant ions—Fe^3^^+^ and Zn^2^^+^ (lattice cations), Ba^2^^+^ (dopant), as well as Pb^2^^+^ and Cd^2^^+^—were also evaluated. Their presence at elevated concentrations did not cause significant suppression of Cu­(II) recovery, confirming that self-exchange or site-blocking effects were negligible under the studied conditions.

Consistent with competitive adsorption on ferrite surfaces, the modest decrease in Cu­(II) recovery observed in the presence of Ba^2^^+^, Fe^3^^+^, and Zn^2^^+^ at deliberately high spike levels (2.0 mg L^–^^1^) is attributed to transient site competition and ionic-strength effects; importantly, recoveries remained ≥83.5%, which is acceptable for worst-case selectivity testing and exceeds typical environmental concentrations of these ions.^48^

These findings underscore the high selectivity and robustness of the proposed method, which may be associated with the strong affinity of the nanosorbent for Cu­(II) through coordination interactions, even in the presence of competing ions. The adsorption behavior is consistent with the known tendency of Cu­(II) to form stable surface complexes, whose ionic radius and coordination preferences could influence.

## 4 Conclusions

A novel and efficient *d*-SPE-FAAS method based on Ba-doped ZnFe_2_O_4_ spinel nanoparticles was successfully developed for the selective preconcentration and FAAS-based quantification of Cu­(II) ions in complex matrices. The synthesis approach yielded structurally pure and surface-active nanoparticles, as confirmed by XRD, FTIR, SEM, and EDX analyses. Optimization of critical analytical parameters revealed that maximum recovery was achieved under mildly alkaline conditions (pH 9.0) using 40 mg of sorbent, 0.3 mL of concentrated HNO_3_ as the eluent, and 60 s of vortex mixing for adsorption and ultrasonic treatment for desorption. The method provided a linear response over the range of 5.0–300 ng mL^–^^1^ with a high correlation coefficient (*R* ^2^ = 0.9992), low LOD (0.67 ng mL^–^^1^), and LOQ (2.23 ng mL^–^^1^), along with an LOD improvement factor of 62 and an RSD of 5.5%. In addition, a simple Langmuir isotherm analysis at pH 9.0 confirmed a maximum capacity of 104.2 mg g^–^^1^, further substantiating the material’s strong binding performance.

Recovery experiments in tap water, green tea infusion, and municipal wastewater yielded excellent results (94.2–105.6%), confirming the method’s robustness and accuracy in diverse sample types. Interference studies demonstrated that commonly encountered cations and anions had a negligible impact on Cu­(II) recovery, highlighting the high selectivity of the nanosorbent. Matrix-matched calibration and optimized pretreatment steps further ensured reliable quantification in real-world applications.

Compared with existing *d*-SPE and SPE protocols in the literature, the proposed method stands out for its simplicity, cost-efficiency, and excellent sensitivity without requiring sophisticated instrumentation or elaborate modification steps. These features render the Ba-doped ZnFe_2_O_4_-based *d*-SPE-FAAS protocol a promising candidate for routine monitoring of Cu­(II) in environmental and food samples.

## Data Availability

All data supporting the findings of this study are presented within the article. Further methodological details are available from the corresponding author upon reasonable request.
